# Strategies to Identify and Reach Young Women Who Sell Sex With HIV Prevention and Care Services: Lessons Learnt From the Implementation of DREAMS Services in Two Cities in Zimbabwe

**DOI:** 10.2196/32286

**Published:** 2022-07-27

**Authors:** Sungai T Chabata, Rumbidzo Makandwa, Bernadette Hensen, Phillis Mushati, Tarisai Chiyaka, Sithembile Musemburi, Joanna Busza, Sian Floyd, Isolde Birdthistle, James R Hargreaves, Frances M Cowan

**Affiliations:** 1 Centre for Sexual Health and HIV/AIDS Research (CeSHHAR) Harare Zimbabwe; 2 Department of Public Health Erasmus MC University Medical Center Rotterdam Rotterdam Netherlands; 3 Department of Clinical Research London School of Hygiene and Tropical Medicine London United Kingdom; 4 Centre for Evaluation London School of Hygiene and Tropical Medicine London United Kingdom; 5 Department of Infectious Disease Epidemiology London School of Hygiene and Tropical Medicine London United Kingdom; 6 Department of Population Health London School of Hygiene and Tropical Medicine London United Kingdom; 7 Department of International Public Health Liverpool School of Tropical Medicine Liverpool United Kingdom

**Keywords:** respondent-driven sampling, peer outreach, female sex worker, young women who sell sex, HIV prevention, Zimbabwe, sub-Saharan Africa

## Abstract

**Background:**

Young women who sell sex (YWSS), are underserved by available HIV prevention and care services. The Determined, Resilient, Empowered, AIDS-free, Mentored and Safe (DREAMS) Partnership aimed to reduce the risk of HIV acquisition among vulnerable populations of adolescent girls and young women, including YWSS, in 10 sub-Saharan African countries. We describe 2 methods, respondent-driven sampling (RDS) and peer outreach, used to refer YWSS for DREAMS services in Zimbabwe, and compare the characteristics and engagement of YWSS referred to these services by each method. We hypothesized that RDS would identify YWSS at higher risk of HIV and those who were less engaged with HIV prevention and care services than peer outreach.

**Objective:**

We aimed to compare respondent-driven sampling and peer outreach in recruiting and referring high-risk populations for HIV prevention and care services.

**Methods:**

We used RDS, a sampling method designed to reach a representative sample of the network of key populations, and peer outreach, a programmatic approach to identify, reach, and refer YWSS for DREAMS between April and July 2017, and January 2017 and July 2018, respectively, in 2 cities in Zimbabwe. For RDS, we conducted detailed mapping to understand sex work typology and geography, and then purposively selected 10 “seed” participants in each city to initiate RDS. For peer outreach, we initiated recruitment through 18 trained and age-matched peer educators using youth-tailored community mobilization. We described the characteristics and service engagement of YWSS who accessed DREAMS services by each referral approach and assessed the association of these characteristics with referral approach using the chi-square test. Analysis was performed with and without restricting the period when RDS took place. We estimated the relative incremental costs of recruiting YWSS using each strategy for referral to DREAMS services.

**Results:**

Overall, 5386 and 1204 YWSS were referred for DREAMS services through peer outreach and RDS, respectively. YWSS referred through RDS were more likely to access DREAMS services compared to YWSS referred through peer outreach (501/1204, 41.6% vs 930/5386, 17.3%; *P*<.001). Regardless of referral approach, YWSS who accessed DREAMS had similar education levels, and a similar proportion tested HIV negative and reported not using a condom at the last sex act. A higher proportion of YWSS accessing DREAMS through RDS were aged 18-19 years (167/501, 33.3% vs 243/930, 26.1%; *P*=.004) and more likely to be aware of their HIV status (395/501, 78.8% vs 396/930, 42.6%; *P*<.001) compared to those accessing DREAMS services through peer outreach. The incremental cost per young woman who sells sex recruited was US $7.46 for peer outreach and US $52.81 for RDS.

**Conclusions:**

Peer outreach and RDS approaches can reach and refer high-risk but different groups of YWSS for HIV services, and using both approaches will likely improve reach.

**International Registered Report Identifier (IRRID):**

RR2-DOI 10.1186/s12889-018-5085-6

## Introduction

Achieving the ambitious goal to end the AIDS epidemic by 2030 requires that programs identify and reach populations at high risk of HIV. Adolescent girls and young women with high-risk sexual lifestyles, such as young women who sell sex, remain a priority [1-3]. In many countries, including Zimbabwe, selling sex is criminalized, making young women who sell sex particularly hidden and hard to reach with HIV prevention and care services.

Strategies to identify, reach, and engage young women who sell sex are critical. The approaches most commonly used are venue-based approaches, including community-based outreach [4], and network-based approaches, including peer outreach and enhanced peer outreach [5]. Community-based outreach involves the use of peer educators and outreach workers to access and engage with hidden populations in communities where they congregate. Peer outreach is based on the reasoning that by engaging with peers who match the desired population, one may reach unidentified, harder-to-reach, high-risk populations [6]. Enhanced peer outreach is an incentivized version of peer outreach similar in design to respondent-driven sampling (RDS), [7] where outreach workers give coupons to peer mobilizers to recruit other peers within their network. Peer mobilizers are selected from the hidden population based on factors, such as their network size, communication skills, risk behaviors, age, location, and knowledge of peers who engage in high-risk behaviors or have never accessed HIV services [6]. The difference between enhanced peer outreach and RDS is that enhanced peer outreach is a programmatic approach where the number of coupons given to each mobilizer is not restricted, while RDS is a sampling method designed to recruit a representative sample of the network of the population in question, and thus, the number of coupons given to each recruiter is limited to reduce the likelihood of overrepresentation of participants with more extensive networks [7].

Between 2016 and 2019, the Determined, Resilient, Empowered, AIDS-free, Mentored, and Safe (DREAMS) Partnership aimed to reduce the risk of HIV acquisition among the most vulnerable adolescent girls and young women, including young women who sell sex, in 10 sub-Saharan African countries by providing a package of biomedical, social, and economic interventions [8,9]. To ensure that young women who sell sex were reached during implementation of the DREAMS Partnership in Zimbabwe, we used peer outreach, a programmatic strategy to identify and refer young women who sell sex for DREAMS services. Separately, we used RDS, a research sampling strategy, to recruit young women who sell sex to a research cohort to determine the impact of DREAMS on HIV incidence, with cohort participants offered onward referral to DREAMS services [3,10]. DREAMS was implemented and the research cohorts were recruited in 2 Zimbabwean cities. Here, we compared the characteristics and engagement of young women who sell sex, who were referred to DREAMS services for young women who sell sex [11], using each method. We assessed which strategy reached more young women who sell sex, hypothesizing that RDS would identify young women who sell sex at higher risk of HIV and who were less engaged with HIV prevention and care services than peer outreach.

## Methods

### The DREAMS Partnership

The DREAMS partnership offered a comprehensive package of evidence-based social and clinical interventions to reduce the rate of new HIV infections and other outcomes among adolescent girls and young women. In Zimbabwe, DREAMS was implemented in 6 districts in Bulawayo, Chipinge, Gweru, Makoni, Mazowe, and Mutare [12]. Six implementing partners delivered the DREAMS package, which included social protection services, gender-based violence prevention and care services, and HIV prevention services, including an offer of pre-exposure prophylaxis to young women who sell sex aged 18 to 24 years. The overall coordination of DREAMS implementing partners differed by region. To increase economic well-being, young women who sell sex were offered economic empowerment programs, including vocational skills training, financial literacy training, savings and lending support, and services to support access to education, including cash transfers and educational subsidies.

### The Sisters With a Voice Program

DREAMS services for young women who sell sex were accessible through the Sisters with a Voice program (Sisters), in addition to other implementing partners. As described elsewhere [11,13], Sisters is a national sex worker program that was established in Zimbabwe in 2009 on behalf of the Ministry of Health and Child Care, and the National AIDS Council. Briefly, Sisters provides free condoms and contraception, HIV testing and counseling, syndromic management of sexually transmitted infections, health education, and legal advice supported by a network of peer educators.

### Study Location and Participants

This study was conducted in 2 districts/cities (Bulawayo and Mutare) that were selected as part of DREAMS impact evaluation from the 6 districts where DREAMS was being implemented [9]. A cohort of young women who sell sex was recruited using RDS and followed-up for 24 months to determine the impact of DREAMS on HIV incidence among young women who sell sex in these 2 cities compared to the incidence among young women who sell sex in 4 towns where DREAMS was not implemented and young women who sell sex only had access to Sisters services [9,10]. Young women who sell sex were defined as adolescent girls and young women aged 18 to 24 who reported exchanging sex with someone because they gave them money, a gift, or material support (important was that sex would not have happened without that exchange). Young women who sell sex included those who self-identified as female sex workers or not. Informal discussions about sex work environment at each location were conducted to ensure that only young women who sell sex were recruited in the study.

### Recruiting Young Women Who Sell Sex for DREAMS Services

#### Peer Outreach

We identified and enrolled young women who sell sex to DREAMS services through a network of 18 (9 in each city) trained and age-matched peer educators supported and directly supervised by the Sisters program outreach team. Peer educators received a monthly incentive of US $15. As described in detail elsewhere [1], peer educators are trained to build rapport with other young women who sell sex and mobilize them for HIV prevention and care services, using a range of community mobilization activities and materials that were specifically tailored for reaching young women who sell sex aged <25 years. Community mobilization materials included 21 activity packs, organized into 6 modules relating to social asset building, HIV prevention, condom promotion and provision, gender norms, basic financial literacy, and sexual violence prevention [1]. These materials were designed in consultation with young women who sell sex and experts, and were piloted and evaluated among a sample of young women who sell sex using in-depth interviews and focus group discussions [1].

Peer educators were recruited in 2016 and worked in the DREAMS program until 2018. Peer educators included 10 who self-identified as female sex workers and 8 who did not. All were 18 to 20 years old and had some secondary school education or higher. Their duties involved identifying hotspots and adolescent girls and young women through word of mouth. Hotspots included secondary schools, colleges, streets, parties, bars, and homes. Peer educators would meet with young women, talk to them about the nature of DREAMS services and where they could access the services, and, if they were interested, give them a referral slip to be produced the first time a young woman who sells sex accessed DREAMS services. Peer outreach was conducted between January 2017 and July 2018.

#### Respondent-Driven Sampling

For the evaluation cohort, we recruited young women who sell sex, using RDS. First, we conducted detailed social mapping as described elsewhere [14]. A team of field workers was trained to engage with young women who sell sex at different locations whether they self-identified as female sex workers or not [14]. They visited hotspots where young women who sell sex were likely to solicit clients as informed by discussions with local peer educators, selecting the 3 busiest days of the week, Thursday, Friday, and Saturday, over 2 consecutive weekends [14]. During site visits, the research team used direct observation, group discussions, and informal interviews.

Mapping helped to understand sex work typology and geography, and was used to purposively select 10 “seed” participants to initiate RDS recruitment at each site. Seeds represented each identified sex work typology, geography, and target age group. Of the 20 seeds selected across the 2 cities, 80% (16/20) were 20 to 24 years old, 95% (19/20) had some secondary school education or higher, 70% (14/20) were single or never married, and 75% (15/20) self-identified as female sex workers. Young women who sell sex were eligible to participate in RDS surveys if they were aged 18 to 24 years. Each “seed” provided written informed consent to participate, and was interviewed and given 2 recruitment coupons to pass on to young women who sell sex in her social network [9]. Young women who sell sex, who received a coupon attended the survey site, provided written informed consent to participate, and, on completion of survey activities, were given 2 coupons to pass on to 2 further young women who sell sex they knew, who sold sex in that location and who had not previously been recruited to the survey. The recruitment process happened over 6 waves until the desired sample size of about 600 young women who sell sex was achieved in each city [9]. All young women who sell sex were referred for DREAMS services. Importantly, recruitment through RDS (but not peer outreach) was incentivized; young women who sell sex were given an incentive of US $3 for participating in the survey themselves, and an additional US $2 for each peer recruited [9]. RDS was conducted for 4 months, from April to July 2017.

Key similarities between the approaches were that both were network-based and started with a purposive sample of peers for peer outreach or seeds for RDS. However, for peer outreach, peer educators received a week of training, were supported by a network of outreach workers, and had a host of materials to support mobilization activities. Their relationship with the peers they recruited was intended to be ongoing. Recruitment through RDS was limited to 2 recruits per recruiter, and participation was incentivized [9]. Seeds received a short script of what to say when passing coupons on but not formal training and were not expected to continue their engagement with the young women who sell sex.

#### Unique Identifiers

Both DREAMS program beneficiaries and survey participants were assigned an alphanumeric identifier that comprised the first letter of the first name, the last 3 letters of the surname, and the date of birth to link young women who sell sex across DREAMS services. We deidentified peer outreach and Sisters point of access data, and used the alphanumeric identifier to establish if young women who sell sex had engaged with DREAMS services through the Sisters program.

### Statistical Analyses

Participants included in these analyses were young women who sell sex aged 18 to 24 years, who were reached in the 2 cities where both approaches were conducted. Of note, there were no age restrictions for young women who sell sex referred for DREAMS services through peer outreach, but analyses were restricted to young women who sell sex, who were aged 18 to 24 years to match RDS data. We described the sociodemographic characteristics of young women who sell sex recruited into the RDS survey by city and compared the characteristics across the 2 cities. RDS data were RDS-II weighted, with women’s responses weighted by the inverse of the reported number of young women who sell sex that they knew, that is, the number of other young women who sell sex that they could have recruited to the survey. Description of sociodemographic characteristics of peer outreach data was not possible owing to limited data routinely captured at the time of program referral.

Among young women who sell sex, who accessed DREAMS services, we described the demographic characteristics, sexual behaviors, and previous service engagement by referral approach and assessed if these differed by referral approach, using the chi-square test. Analysis was performed regardless of the referral period. For peer outreach, we restricted the comparison to the period when RDS took place. Young women who sell sex referred through both approaches were retained in the analysis since excluding them did not make any difference to the results.

Subsequently, we used logistic regression to identify sociodemographic characteristics and sexual behaviors associated with ever access of DREAMS services among young women who sell sex, who were recruited through RDS. For logistic regression analyses, seed participants were dropped. Factors associated with access of DREAMS services at *P*≤.10 in univariable analysis were included in the multivariable regression model, adjusting for all factors associated with access of DREAMS services in the univariable analysis. Again, this was not possible for young women who sell sex, who accessed services through peer outreach owing to limited point of referral data.

Finally, we estimated the relative incremental costs of recruiting young women who sell sex using each strategy for referral to DREAMS services.

### Ethics Approval

The DREAMS impact evaluation was reviewed and approved by the Medical Research Council of Zimbabwe (MRCZ/A/2085) and the London School of Hygiene and Tropical Medicine (11835). All participants were given information about the study and were asked for written informed consent for participation.

## Results

### Characteristics of Young Women Who Sell Sex Recruited Through RDS

Between April and July 2017, 1204 young women who sell sex were recruited to RDS surveys and referred for DREAMS services in the 2 cities. Among these young women who sell sex, the majority were aged 20 to 24 years (799/1204, 64.6%), had some or complete secondary school education (1079/1204, 88.2%), were never married (801/1204, 67.9%), and self-identified as female sex workers (801/1191, 65.0%) (Table 1). Young women who sell sex in Bulawayo were less likely to be divorced or separated (75/601, 12.2% vs 290/603, 44.8%), were less likely to self-identify as female sex workers (367/590, 60.1% vs 434/601, 69.7%), and were more likely to report having more than six alcoholic drinks in 1 night during the last 12 months (277/601, 42.5% vs 138/602, 19.9%), compared to those in Mutare. Additionally, young women who sell sex from Bulawayo were less likely to report condom use at the last sex act with a regular partner (275/476, 57.8% vs 312/431, 74.3%) or client (419/490, 85.0% vs 422/456, 92.0%) compared to young women who sell sex in Mutare. A higher proportion of young women who sell sex from Bulawayo reported condom-less sex with a regular partner (246/477, 54.0% vs 164/431, 33.8%) or client (98/491, 20.4% vs 65/458, 14.1%) in the past month compared to those from Mutare. Overall, almost a quarter (471/1204, 37.8%) of young women who sell sex reported being at risk of common mental disorders within the last week.

**Table 1:** Sociodemographic and sexual behavioral characteristics of young women who sell sex recruited to respondent-driven sampling (RDS) surveys in 2 Zimbabwean cities (RDS-II weighted; N=1204).

Characteristic		Bulawayo (N=601), n/N (%)	Mutare (N=603), n/N (%)	Total (N=1204), n/N (%)	*P* value
**Age at recruitment (years)**					.04
	18-19	222/601 (38.8)	183/603 (32.0)	405/1204 (35.4)	
	20-24	379/601 (61.2)	420/603 (68.0)	799/1204 (64.6)	
**Highest level of education**					.36
	None/incomplete primary	12/601 (2.6)	29/603 (4.6)	41/1204 (3.6)	
	Complete primary	49/601 (8.9)	35/603 (7.4)	84/1204 (8.1)	
	Incomplete secondary	245/601 (40.4)	277/603 (43.2)	522/1204 (41.8)	
	Complete secondary/higher	295/601 (48.1)	262/603 (44.8)	557/1204 (46.4)	
**Marital status**					<.001
	Single/never married	501/601 (83.2)	300/603 (52.8)	801/1204 (67.9)	
	Married/living together as if married	22/601 (4.3)	9/603 (1.8)	31/1204 (3.1)	
	Divorced/separated	75/601 (12.2)	290/603 (44.8)	365/1204 (28.5)	
	Widowed	3/601 (0.2)	4/603 (0.7)	7/1204 (0.5)	
**Self-identification as a sex worker**					.004
	No	223/590 (39.9)	167/601 (30.3)	390/1191 (35.0)	
	Yes	367/590 (60.1)	434/601 (69.7)	801/1191 (65.0)	
**Age at start of selling sex (years)**					.01
	≤15	86/600 (13.2)	60/603 (9.2)	146/1203 (11.2)	
	16-17	222/600 (37.2)	190/603 (31.6)	412/1203 (34.4)	
	18-24	292/600 (49.6)	353/603 (59.1)	645/1203 (54.4)	
**Years selling sex**					.02
	0-2	291/600 (52.2)	323/603 (55.9)	614/1203 (54.1)	
	3-4	204/600 (32.7)	150/603 (24.8)	354/1203 (28.7)	
	≥5	105/600 (15.1)	130/603 (19.3)	235/1203 (17.2)	
**Number of sexual partners in the past month**					.07
	0-4	272/601 (50.4)	265/603 (47.8)	537/1204 (49.1)	
	5-9	159/601 (24.7)	127/603 (20.6)	286/1204 (22.6)	
	≥10	170/601 (24.9)	211/603 (31.6)	381/1204 (28.3)	
**Number of clients in the past month**					.08
	0-4	313/601 (57.5)	296/603 (53.6)	609/1204 (55.5)	
	5-9	131/601 (20.0)	110/603 (17.4)	241/1204 (18.7)	
	≥10	157/601 (22.5)	197/603 (29.0)	354/1204 (25.8)	
**Condom use at the last sex act with a regular partner**					<.001
	No	201/476 (42.2)	119/431 (25.7)	320/907 (34.3)	
	Yes	275/476 (57.8)	312/431 (74.3)	587/907 (65.7)	
**Condom-less sex with a regular partner in the past month**					<.001
	No	231/477 (46.0)	267/431 (66.2)	498/908 (55.7)	
	Yes	246/477 (54.0)	164/431 (33.8)	410/908 (44.3)	
**Condom use at the last sex act with a client**					.006
	No	71/490 (15.0)	34/456 (8.0)	105/946 (11.6)	
	Yes	419/490 (85.0)	422/456 (92.0)	841/946 (88.4)	
**Condom-less sex with a client in the past month**					.03
	No	393/491 (79.6)	393/458 (85.9)	786/949 (82.6)	
	Yes	98/491 (20.4)	65/458 (14.1)	163/949 (17.4)	
**STI^a^ symptoms in the last 12 months**					.17
	No	470/601 (78.7)	449/603 (74.8)	919/1204 (76.7)	
	Yes	131/601 (21.3)	154/603 (25.2)	285/1204 (23.3)	
**Accessed DREAMS^b^ services**					<.001
	No	277/601 (46.2)	426/603 (69.6)	703/1204 (57.9)	
	Yes	324/601 (53.8)	177/603 (30.4)	501/1204 (42.1)	
**Relationship with other young women who sell sex**					.10
	Good	450/600 (72.6)	464/601 (76.0)	914/1201 (74.3)	
	Neither good nor bad	102/600 (19.3)	81/601 (14.0)	183/1201 (16.7)	
	No relation	48/600 (8.1)	56/601 (10.0)	104/1201 (9.0)	
**Number of close friends**					.02
	0	85/601 (16.2)	133/603 (22.8)	218/1204 (19.5)	
	≥1	516/601 (83.8)	470/603 (77.2)	986/1204 (80.5)	
**Binge drinking^c^**					<.001
	No alcohol in the last 12 months	153/601 (29.4)	248/602 (44.8)	401/1203 (37.1)	
	Drank alcohol but no occasions of binge drinking	171/601 (28.1)	216/602 (35.3)	387/1203 (31.7)	
	Yes, at least one occasion of binge drinking	277/601 (42.5)	138/602 (19.9)	415/1203 (31.2)	
**Risk of CMD^d^**					.73
	No	366/601 (61.6)	367/603 (62.7)	733/1204 (62.2)	
	Yes	235/601 (38.4)	236/603 (37.3)	471/1204 (37.8)	
**Experienced any form of violence from a sexual partner**					<.001
	No	351/601 (60.8)	280/603 (49.2)	631/1204 (55.0)	
	Yes	250/601 (39.2)	323/603 (50.8)	573/1204 (45.0)	
**Experienced any form of violence from police**					.26
	No	576/601 (96.5)	568/602 (95.1)	1144/1203 (95.8)	
	Yes	25/601 (3.5)	34/602 (4.9)	59/1203 (4.2)	

^a^STI: sexually transmitted infection.

^b^DREAMS: Determined, Resilient, Empowered, AIDS-free, Mentored, and Safe.

^c^Had more than six alcoholic drinks in 1 night during the last 12 months.

^d^CMD: common mental disorder.

### Characteristics of Young Women Who Sell Sex, Who Accessed DREAMS Services by Referral Approach

Between January 2017 and July 2018, 5386 young women who sell sex were referred to DREAMS services via peer outreach. Between April and July 2017, 1204 young women who sell sex were referred to DREAMS services via RDS. Young women who sell sex referred through RDS were over twice as likely to access DREAMS services through the Sisters program (501/1204, 41.6%) compared to young women who sell sex referred through peer outreach (930/5386, 17.3%; *P*<.001). Additionally, 45 young women who sell sex, 5% (45/930) of those who accessed DREAMS via peer outreach and 9% (45/501) of those who accessed DREAMS via RDS, were referred through both strategies. Services accessed included HIV testing, free condoms and contraception, and other services such as sexually transmitted infection treatment.

Between April and July 2017, when the RDS was ongoing, 1228 young women who sell sex were referred through peer outreach. When restricting our comparison to the period when RDS was ongoing, young women who sell sex referred through RDS remained more likely to access DREAMS services (501/1204, 41.6%) compared to young women who sell sex referred through peer outreach (212/1228, 17.3%; *P*<.001). Within this period, 3.0% (15/501) of those who accessed DREAMS via RDS and 7.1% (15/212) of those who accessed DREAMS via peer outreach were referred through both strategies.

A higher proportion of young women who sell sex accessing DREAMS services through RDS were younger (18-19 years old: 167/501, 33.3% vs 243/930, 26.1%; *P*=.004) and reported having ever been tested for HIV (441/501, 88.0% vs 661/827, 79.9%; *P*<.001) compared to young women who sell sex accessing DREAMS services through peer outreach (Table 2). Additionally, young women who sell sex accessing DREAMS services through RDS were more likely to self-report an HIV-positive status (39/439, 8.9% vs 0/661, 0.0%; *P*<.001), more likely to be aware of their HIV status (395/501, 78.8% vs 396/930, 42.6%; *P*<.001), and less likely to report no condom use at the last sex act with any partner (158/501, 31.5% vs 313/775, 40.4%; *P*=.001) compared to those accessing DREAMS services through peer outreach.

Similar proportions of young women who sell sex had completed some secondary school or higher (456/501, 91.0% vs 788/873, 90.3%; *P*=.65) and tested HIV positive as part of the RDS survey or through the Sisters program (76/499, 15.2% vs 123/745, 16.5%; *P*=.55; Table 2). Restricting to the period when RDS was ongoing did not change the results, except that the age distribution of the young women who sell sex was similar regardless of referral approach (18-19 years old: 167/501, 33.3% vs 61/212, 28.8%; *P*=.23; Table 3).

**Table 2:** Comparison of the characteristics of young women who sell sex, who accessed Determined, Resilient, Empowered, AIDS-free, Mentored, and Safe (DREAMS) services through the Sisters program by referral approach.

Characteristic			Accessed DREAMS^a^ through peer outreach (N=930), n/N (%)		Accessed DREAMS through RDS^b^ referral (N=501), n/N (%)		*P* value
**Age (years)**							.004
	18-19	243/930 (26.1)		167/501 (33.3)			
	20-24	687/930 (73.9)		334/501 (66.7)			
**Educational attainment**							.65
	Primary school or less	85/873 (9.7)		45/501 (9.0)			
	Some secondary school or more	788/873 (90.3)		456/501 (91.0)			
**City**							<.001
	Bulawayo	466/930 (50.1)		324/501 (64.7)			
	Mutare	464/930 (49.9)		177/501 (35.3)			
**Ever tested for HIV**							<.001
	No	166/827 (20.1)		60/501 (12.0)			
	Yes	661/827 (79.9)		441/501 (88.0)			
**Reported HIV status before contact with RDS or peer outreach^c^**							<.001
	Negative	661/661 (100.0)		400/439 (91.1)			
	Positive	0/661 (0.0)		39/439 (8.9)			
**HIV status at contact with RDS or peer outreach**							.55
	Negative	622/745 (83.5)		423/499 (84.8)			
	Positive	123/745 (16.5)		76/499 (15.2)			
**Aware of HIV status at contact with RDS or peer outreach^d^**							<.001
	No	534/930 (57.4)		106/501 (21.2)			
	Yes	396/930 (42.6)		395/501 (78.8)			
**No condom used at the last sex act with any partner**							.001
	No	462/775 (59.6)		343/501 (68.5)			
	Yes	313/775 (40.4)		158/501 (31.5)			

^a^DREAMS: Determined, Resilient, Empowered, AIDS-free, Mentored, and Safe.

^b^RDS: respondent-driven sampling.

^c^Among young women who sell sex reporting being ever tested for HIV.

^d^Proportion ever testing HIV positive or having an HIV-negative test during the past 12 months.

**Table 3:** Comparison of the characteristics of young women who sell sex, who were referred during the period when respondent-driven sampling was ongoing and accessed Determined, Resilient, Empowered, AIDS-free, Mentored, and Safe (DREAMS) services through the Sisters program by referral approach.

Characteristic			Accessed DREAMS^a^ through peer outreach (N=212), n/N (%)		Accessed DREAMS through RDS^b^ referral (N=501), n/N (%)		*P* value
**Age (years)**							.23
	18-19	61/212 (28.8)		167/501 (33.3)			
	20-24	151/212 (71.2)		334/501 (66.7)			
**Educational attainment**							.16
	Primary school or less	12/207 (5.8)		45/501 (9.0)			
	Some secondary school or more	195/207 (94.2)		456/501 (91.0)			
**City**							<.001
	Bulawayo	91/212 (42.9)		324/501 (64.7)			
	Mutare	121/212 (57.1)		177/501 (35.3)			
**Ever tested for HIV**							<.001
	No	49/204 (24.0)		60/501 (12.0)			
	Yes	155/204 (76.0)		441/501 (88.0)			
**Reported HIV status before contact with RDS or peer outreach^c^**							<.001
	Negative	155/155 (100.0)		400/439 (91.1)			
	Positive	0/155 (0.0)		39/439 (8.9)			
**HIV status at contact with RDS or peer outreach**							.63
	Negative	175/203 (86.2)		423/499 (84.8)			
	Positive	28/203 (13.8)		76/499 (15.2)			
**Aware of HIV status at contact with RDS or peer outreach^d^**							<.001
	No	107/212 (50.5)		106/501 (21.2)			
	Yes	105/212 (49.5)		395/501 (78.8)			
**No condom used at the last sex act with any partner**							.01
	No	118/201 (58.7)		343/501 (68.5)			
	Yes	83/201 (41.3)		158/501 (31.5)			

^a^DREAMS: Determined, Resilient, Empowered, AIDS-free, Mentored, and Safe.

^b^RDS: respondent-driven sampling.

^c^Among young women who sell sex reporting being ever tested for HIV.

^d^Proportion ever testing HIV positive or having an HIV-negative test during the past 12 months.

### Factors Associated With Access of DREAMS Services in the RDS Sample

In adjusted analyses, there was strong evidence that access to DREAMS services was lower among young women who sell sex from Mutare compared to those from Bulawayo (177/603, 30.4% vs 324/601, 53.8%; adjusted OR [aOR] 0.37, 95% CI 0.28-0.50; *P*<.001; Table 4). Young women who sell sex, who started selling sex at 16 to 17 years (183/412, 45.5% vs 46/146, 30.1%; aOR 2.39, 95% CI 1.32-4.32; *P*=.004) or 18 to 24 years (271/645, 42.4% vs 46/146, 30.1%; aOR 2.42, 95% CI 1.36-4.29; *P*=.003) were more likely to access DREAMS services compared to those who started selling sex at 15 years or less. There was evidence that young women who sell sex, who reported at least one close friend were more likely to access DREAMS services compared to those who did not have a close friend (425/986, 44.0% vs 76/218, 34.3%; aOR 1.62, 95% CI 1.03-2.53; *P*=.04).

**Table 4:** Factors associated with ever access of Determined, Resilient, Empowered, AIDS-free, Mentored, and Safe (DREAMS) services among young women who sell sex, who were referred through respondent-driven sampling (RDS; RDS-II weighted).

Characteristic		Total (N=1204), n (%)	YWSS^a^ who accessed DREAMS^b^ services via RDS^c^ (N=501), n (%)	Crude OR^d^ (95% CI)	*P* value	Adjusted OR (95% CI)	*P* value
**Age at recruitment (years)**					.75		N/A^e^
	18-19	405 (35.4)	167 (41.4)	1		N/A	
	20-24	799 (64.6)	334 (42.5)	1.05 (0.79-1.39)		N/A	
**Highest level of education**					.01		.87
	Primary school or less	125 (11.7)	45 (33.5)	1		1	
	Incomplete secondary school	522 (41.8)	199 (38.9)	1.26 (0.78-2.04)		1.01 (0.55-1.85)	
	Complete secondary or higher	557 (46.4)	257 (47.2)	1.77 (1.10-2.85)		1.11 (0.60-2.06)	
**City**					<.001		<.001
	Bulawayo	601 (49.9)	324 (53.8)	1		1	
	Mutare	603 (50.1)	177 (30.4)	0.37 (0.28-0.50)		0.43 (0.29-0.62)	
**Marital status**					.001		.94
	Never married	801 (67.9)	366 (46.0)	1		1	
	Ever married	403 (32.1)	135 (33.8)	0.60 (0.44-0.81)		0.98 (0.66-1.47)	
**Self-identification as a sex worker**					.37		N/A
	No	390 (35.0)	177 (44.1)	1		N/A	
	Yes	801 (65.0)	317 (40.8)	0.88 (0.66-1.17)		N/A	
**Age at the start of selling sex (years)**					.02		.008
	≤15	146 (11.2)	46 (30.1)	1		1	
	16-17	412 (34.4)	183 (45.5)	1.93 (1.22-3.07)		2.39 (1.32-4.32)	
	18-24	645 (54.4)	271 (42.4)	1.71 (1.10-2.64)		2.42 (1.36-4.29)	
**Years selling sex**					.20		N/A
	0-2	614 (54.1)	275 (44.1)	1		N/A	
	3-4	354 (28.7)	143 (41.9)	0.92 (0.67-1.26)		N/A	
	≥5	235 (17.2)	82 (36.0)	0.72 (0.50-1.03)		N/A	
**Number of sexual partners in the past month**					.002		.58
	0-4	537 (49.1)	227 (44.0)	1		1	
	5-9	286 (22.6)	141 (48.9)	1.22 (0.87-1.71)		1.21 (0.61-2.38)	
	≥10	381 (28.3)	133 (33.4)	0.64 (0.46-0.88)		0.69 (0.20-2.33)	
**Number of clients in the past month**					.001		.96
	0-4	609 (55.5)	267 (44.1)	1		1	
	5-9	241 (18.7)	114 (49.0)	1.21 (0.85-1.72)		1.09 (0.53-2.27)	
	≥10	354 (25.8)	120 (32.3)	0.60 (0.43-0.83)		0.97 (0.28-3.39)	
**Condom use at the last sex act with a regular partner**					.03		.19
	No	320 (34.3)	155 (49.2)	1		1	
	Yes	587 (65.7)	236 (40.0)	0.69 (0.49-0.95)		0.74 (0.47-1.16)	
**Condom-less sex with a regular partner in the past month**					.03		.92
	No	498 (55.7)	205 (39.3)	1		1	
	Yes	410 (44.3)	186 (47.8)	1.41 (1.03-1.93)		1.02 (0.66-1.57)	
**Condom use at the last sex act with a client**					.52		N/A
	No	105 (11.6)	45 (44.7)	1		N/A	
	Yes	841 (88.4)	350 (40.8)	0.85 (0.53-1.38)		N/A	
**Condom-less sex with a client in the past month**					.26		N/A
	No	786 (82.6)	322 (40.2)	1		N/A	
	Yes	163 (17.4)	74 (46.0)	1.27 (0.84-1.90)		N/A	
**STI^f^ symptoms in the last 12 months**					.07		.96
	No	919 (76.7)	404 (43.8)	1		1	
	Yes	285 (23.3)	97 (36.6)	0.74 (0.53-1.02)		0.99 (0.67-1.47)	
**Relationship with other YWSS**					.19		N/A
	Good	914 (74.3)	379 (42.1)	1		N/A	
	Neither good nor bad	183 (16.7)	84 (47.1)	1.22 (0.84-1.78)		N/A	
	No relation	104 (9.0)	37 (34.1)	0.71 (0.43-1.17)		N/A	
**Number of close friends**					.03		.04
	0	218 (19.5)	76 (34.3)	1		1	
	≥1	986 (80.5)	425 (44.0)	1.51 (1.05-2.17)		1.62 (1.03-2.53)	
**Binge drinking^g^**					.046		.44
	No alcohol in the last 12 months	401 (37.1)	156 (38.6)	1		1	
	Drank alcohol but no occasions of binge drinking	387 (31.7)	153 (40.1)	1.06 (0.76-1.49)		1.18 (0.78-1.77)	
	Yes, at least one occasion of binge drinking	415 (31.2)	191 (48.2)	1.48 (1.06-2.06)		1.32 (0.86-2.03)	
**Risk of CMD^h^**					.93		N/A
	No	733 (62.2)	296 (42.0)	1		N/A	
	Yes	471 (37.8)	205 (42.3)	1.01 (0.77-1.34)		N/A	
**Experienced any form of violence from a sexual partner**					.009		.13
	No	631 (55.0)	284 (46.1)	1		1	
	Yes	573 (45.0)	217 (37.2)	0.69 (0.53-0.91)		0.76 (0.54-1.08)	
**Experienced any form of violence from police**					.95		N/A
	No	1144 (95.8)	476 (42.1)	1		N/A	
	Yes	59 (4.2)	24 (42.6)	1.02 (0.56-1.86)		N/A	

^a^YWSS: young women who sell sex.

^b^DREAMS: Determined, Resilient, Empowered, AIDS-free, Mentored, and Safe.

^c^RDS: respondent-driven sampling.

^d^OR: odds ratio.

^e^N/A: not applicable.

^f^STI: sexually transmitted infection.

^g^Had more than six alcoholic drinks in 1 night during the last 12 months.

^h^CMD: common mental disorder.

### Incremental Costs and Requirements of Recruitment Strategies

Peer outreach costs included the cost of formative work to select peer educators that was done in 3 days by a team of 3 program staff members in each city, cost of outreach support, and cost of peer educator incentives for 19 months (Table 5). RDS costs included the cost of formative work to select seed participants that was done in 3 days by a team of 3 research staff members in each city, cost of the RDS survey team (5 staff) who spent 50 days in Bulawayo and 60 days in Mutare, and cost of participant recruitment incentives. The incremental cost per young woman who sells sex recruited was US $7.46 for peer outreach and US $52.81 for RDS.

**Table 5:** Comparison of peer outreach and respondent-driven sampling requirements and incremental costs of recruiting young women who sell sex using each strategy for referral to Determined, Resilient, Empowered, AIDS-free, Mentored, and Safe (DREAMS) services in 2 cities.

Variable		Peer outreach	RDS^a^
**Characteristic**			
	Peer referral	Yes	Yes
	Number of peers/seeds in 2 cities	18	20
	Duration of recruitment (months)	19	4
	Recruitment incentive	No	Yes
**Recruitment costs (US$)^b^**			
	Cost of formative work to identify peers/seeds (per diem)	1350^c^	1350^d^
	Cost of peer educator training and materials	5000	N/A^e^
	Cost of the outreach support team (salaries)^f^	28,500	N/A
	Cost of the RDS survey team (salaries and per diem)^g^	N/A	56,250
	Cost of peer educator monthly incentives^h^	5310	N/A
	Recruitment incentives^i^	N/A	5980
Total cost (US$)		40,160	63,580
Number of young women who sell sex recruited		5386	1204
Cost per young woman who sells sex recruited (US$)		7.46	52.81

^a^RDS: respondent-driven sampling.

^b^Some of the RDS costs that are research specific, such as cost of laboratory testing, have been omitted, and we have only focused on those that are recruitment specific.

^c^3 program staff members in 2 teams at US $75 per diem for 3 days.

^d^3 research staff members in 2 teams at US $75 per diem for 3 days.

^e^N/A: not applicable.

^f^2 outreach workers for 19 months at US $750 salary per month.

^g^5 research assistants for 4 months at US $750 salary per month + 5 research assistants at US $75 per diem for 110 days.

^h^18 peer educators at US $15 monthly incentive for 19 months.

^i^1204 participants at US $3 participant incentive + 1184 recruits at US $2 peer recruitment incentive.

## Discussion

### Principal Findings

In this study, we compared 2 recruitment strategies that focused on identifying and reaching young women who sell sex with DREAMS services in 2 cities in Zimbabwe. Our study suggested that peer referral, whether through RDS or peer outreach, can identify high-risk and underserved young women and refer them to services. Peer outreach was able to identify a higher proportion of young women who have never been tested for HIV and are therefore not aware of their HIV status. RDS was able to refer more young women who sell sex in a short period of time and refer younger women aged 18 to 19 compared to peer outreach. Restricting our analysis to the same period of recruitment, we found that peer outreach referred a higher proportion of young women who sell sex, who were not aware of their HIV status compared to RDS, but the ages of these women were similar regardless of referral approach. Among women who accessed DREAMS services, those referred by RDS were younger and appeared to be better engaged with services, and more women had previously tested for HIV and knew their status. By contrast, peer outreach identified more young women who sell sex, who had never been tested for HIV and were unaware of their HIV status. Although the differences in ever testing between recruitment approaches were not significant (76% vs 88%), when programs are aiming to ensure that all those who are vulnerable are reached, optimizing referral approaches by using a combination of approaches is likely important.

Both referral approaches were successful in reaching young women who sell sex at high risk of HIV, where the majority were HIV negative. In our previous study, we found that HIV prevalence and incidence rise steeply with age among this population [3,15], and thus, supporting young women who sell sex to engage effectively with prevention is critical. Reported noncondom use was high in both groups but even higher among those recruited through peer outreach.

The success of peer referral approaches in reaching high-risk hidden populations has been noted in many populations, including in West and Central Africa where the use of an enhanced (incentivized) peer outreach approach led to increased detection of new HIV-positive key population individuals who would not have been engaged otherwise [5,6]. With their enhanced peer referral approach, the authors were able to reach female sex workers who had not been effectively engaged by routine outreach approaches [5]. Our study, however, demonstrated that a higher proportion of previously unengaged young women who sell sex accessed DREAMS services through peer outreach than through RDS. This is possibly due to the level of training that peer educators received coupled with the tailored community mobilization activities and materials used. The fact that the RDS strategy referred young women who were more likely to already be engaged with HIV services could be because those young women who sell sex were more likely to be visible on the social network of young women who sell sex. Of note, RDS recruitment was done in a limited time frame with participants given a limited number of coupons with the goal of recruiting a representative sample of the network of the population of young women who sell sex, unlike peer outreach where peer educators were expected to recruit as many young women who sell sex as possible in a longer time frame. This may not only have restricted the absolute number of young women who sell sex recruited via RDS but also limited the performance of RDS in reaching less networked young women who sell sex. Nonetheless, our RDS diagnostics reported elsewhere [3] suggested that convergence was achieved in the 2 cities and our sample was likely to be representative of the network of young women who sell sex recruited.

While RDS enrolled young women who sell sex quickly, the requirement to incentivize recruitment at every stage can be costly to integrate into day-to-day programs. We showed that nonincentivized peer outreach is also able to reach high-risk young women who sell sex. Peer outreach provides at least the possibility of the process of referral being associated with longer-term support. Importantly, we found that the different approaches presented here recruited different groups of high-risk women, and the overlap in terms of those recruited was small, even when compared with peer outreach continued over many months. It seems likely that using a combination of approaches will be most effective at optimizing reach and coverage.

Consistent with other findings [16-19], our analysis suggested the importance of a comprehensive, well-coordinated, and scaled-up HIV program in reaching priority populations. Young women who sell sex from Bulawayo, where the DREAMS program was well coordinated with implementing partners working together to build high DREAMS acceptance, were more likely to access DREAMS via RDS compared to those from Mutare. On the other hand, peer outreach performed better in Mutare (a smaller town) where it referred a higher proportion of young women who sell sex than in Bulawayo. Lessons need to be constantly learned between program sites to generate opportunities for program improvement. Moreover, young women who sell sex, who reported at least one close friend were more likely to access DREAMS services compared to those who did not have a close friend, emphasizing the importance of building social cohesion among disempowered communities to optimize their uptake of HIV prevention and care [20-22].

### Limitations

The limitations include the relatively limited data captured routinely at the time of program referral, limiting the possibility of comparing the characteristics of young women who sell sex, who went on to access services with those of young women who did not. Information on the refusal rate and the reason why some young women who sell sex refused to participate in DREAMS may be useful to refine existing recruitment approaches or operationalize novel approaches like starfish sampling that combines time location sampling and RDS [23]. We might not have compared like with like since our RDS, which was done for research purposes, recruited only young women who sell sex, who were 18 years or above, while referrals through peer outreach were able to include younger women who are even more vulnerable.

### Conclusions

Peer outreach and RDS approaches can reach and refer high-risk but different groups of young women who sell sex for HIV services. Use of both these complementary approaches will likely improve reach.

## Abbreviations

aOR: adjusted odds ratio
DREAMS: Determined, Resilient, Empowered, AIDS-free, Mentored, and Safe
RDS: respondent-driven sampling
